# M2 macrophage-derived exosomal *circTMCO3* acts through *miR-515-5p* and ITGA8 to enhance malignancy in ovarian cancer

**DOI:** 10.1038/s42003-024-06095-8

**Published:** 2024-05-16

**Authors:** Xiao-Min Ran, Juan Yang, Zi-Yi Wang, Ling-Zhi Xiao, Yu-Ping Deng, Ke-Qiang Zhang

**Affiliations:** Department of Gynecologic Oncology Ward 5, Hunan Cancer Hospital, The Affiliated Cancer Hospital of Xiangya School of Medicine, Central South University, Changsha, 410013 Hunan Province PR China

**Keywords:** Genetics, Physiology

## Abstract

Tumor-associated macrophages of the M2 phenotype promote cancer initiation and progression. Importantly, M2 macrophage-derived exosomes play key roles in the malignancy of cancer cells. Here, we report that *circTMCO3* is upregulated in ovarian cancer patients, and its high expression indicates poor survival. M2-derived exosomes promote proliferation, migration, and invasion in ovarian cancer, but these effects are abolished by knockdown of *circTMCO3*. Furthermore, *circTMCO3* functions as a competing endogenous RNA for *miR-515-5p* to reduce its abundance, thus upregulating ITGA8 in ovarian cancer. *miR-515-5p* inhibits ovarian cancer malignancy via directly downregulating ITGA8. The decreased oncogenic activity of *circTMCO3*-silencing exosomes is reversed by *miR-515-5p* knockdown or ITGA8 overexpression. Exosomal *circTMCO3* promotes ovarian cancer progression in nude mice. Thus, M2 macrophage-derived exosomes promote malignancy by delivering *circTMCO3* and targeting the *miR-515-5p*/*ITGA8* axis in ovarian cancer. Our findings not only provide mechanistic insights into ovarian cancer progression, but also suggest potential therapeutic targets.

## Abstract (web summary)

This study shows that M2 macrophage-derived exosomes promote malignancy in ovarian cancer cells by delivering circTMCO3 and targeting the miR-515-5p/ITGA8 axis.

## Introduction

Ovarian cancer is one of the most common and fatal gynecological malignancies^1^. Due to the lack of effective early diagnosis strategies, ovarian cancer is typically diagnosed at an advanced stage with low 5-year survival rate of 29%^2^. Platinum-based chemotherapy, such as cisplatin, is one of standard therapies for ovarian cancer^3,4^. However, cisplatin resistance is one of major causes of therapeutic failure. Therefore, elucidating the mechanisms underlying the malignancy of ovarian cancer is crucial for developing therapeutic strategies.

Macrophages are crucial immune cells that can phagocytize bacteria and infected cells, present antigens and trigger inflammation^5^, which are typically divided into classically activated M1 macrophages and alternatively activated M2 macrophages. Importantly, tumor-associated macrophages (TAMs) account for about 50% of tumor mass with M2-polarized phenotypes^6,7^. TAMs are abundant in patients with ovarian cancer^8^, and TAMs promote ovarian cancer progression via regulating immune escape, migration, invasion, and metastasis^9^. However, TAM-mediated regulation of malignancy of ovarian cancer cells remains largely unknown. One of important mechanisms for macrophages is to release exosomes^10^. Exosomes released by M2 macrophages enhance migration and invasion in colon cancer^11^. Exploring macrophage-derived exosome-mediated regulation of ovarian cancer is crucial for a better understanding of the pathogenesis of ovarian cancer.

Circular RNAs (circRNAs) are non-coding RNAs which are formed by back-splicing^12^ and enriched in exosomes^13^. Strikingly, emerging evidence has revealed key roles of exosomal circRNAs in various human cancers. Chen and colleagues found that exosome-derived *circ_0051443* repressed the malignancy of hepatocellular carcinoma (HCC) cells and HCC progression by promoting BAK1 expression^14^. In ovarian cancer, exosomal *circWHSC1* promoted MUC1 expression and peritoneal diffusion and adhesion, contributing to cancer metastasis^15^. Guan et al. found that *circPUM1* facilitated ovarian cancer progression, and exosomal *circPUM1* promoted metastasis^16^. Thus, understanding the mechanisms by which exosomal circRNAs regulate ovarian cancer progression is truly important. A previous study reported increased abundance of *circ_0031017* derived from *TMCO3* gene (also known as *circTMCO3*) in TAM-derived exosomes^17^, suggesting the potential roles of exosomal *circ_0031017* derived from M2 macrophages in cancers. Additionally, *circTMCO3* was upregulated, and it sponged *miR-577* to enhance proliferation, migration, and invasion in gastric cancer^18^. Wang et al. reported that *circTMCO3* was highly expressed in HCC, and *circTMCO3* might represent potentially valuable diagnostic biomarkers for HCC^19^. However, to our knowledge, the roles of *circTMCO3* in cancers remain largely unknown, especially in ovarian cancer. Therefore, we focused the function of M2-derived exosomal *circTMCO3* in ovarian cancer.

One of action mechanisms of circRNAs is that circRNAs function as competitive endogenous RNAs (ceRNAs) to absorb microRNAs (miRNAs) and release inhibitory effects on downstream targets^20^. CircRNAs modulate tumor-related gene expression via sponging miRNAs. *miR-515-5p* works as a tumor suppressor to inhibit proliferation, migration, and metastasis^21^. Integrins are key regulators of cancer growth and metastasis^22^. Integrin subunit alpha 8 (ITGA8) promotes epithelial-mesenchymal transition and cell invasion in multiple myeloma^23^. However, their roles and interaction in ovarian cancer are unknown. We found that *miR-515-5p* had a targeted binding relationship with *circTMCO3* and *ITGA8*, respectively. Therefore, we hypothesized that M2-derived exosomal *circTMCO3* might regulate malignant behaviors in ovarian cancer via sponging *miR-515-5p* and upregulating ITGA8.

To summarize, our investigation aims to study the roles of M2 macrophage-derived exosomes carrying *circTMCO3* in regulating the malignancy of ovarian cancer cells. We found that the increased *circTMCO3* expression was associated the poor survival of patients with ovarian cancer. Further investigation demonstrated that M2 macrophage-derived exosomal *circTMCO3* promoted the malignancy by targeting the *miR-515-5p*/*ITGA8* axis in ovarian cancer. Our findings are beneficial for deepening understanding of the progression of ovarian cancer and providing potential exosome-based therapeutic strategies.

## Results

### Abundant M2 macrophages facilitate ovarian cancer cell proliferation, migration, and invasion

**Fig. 1:**
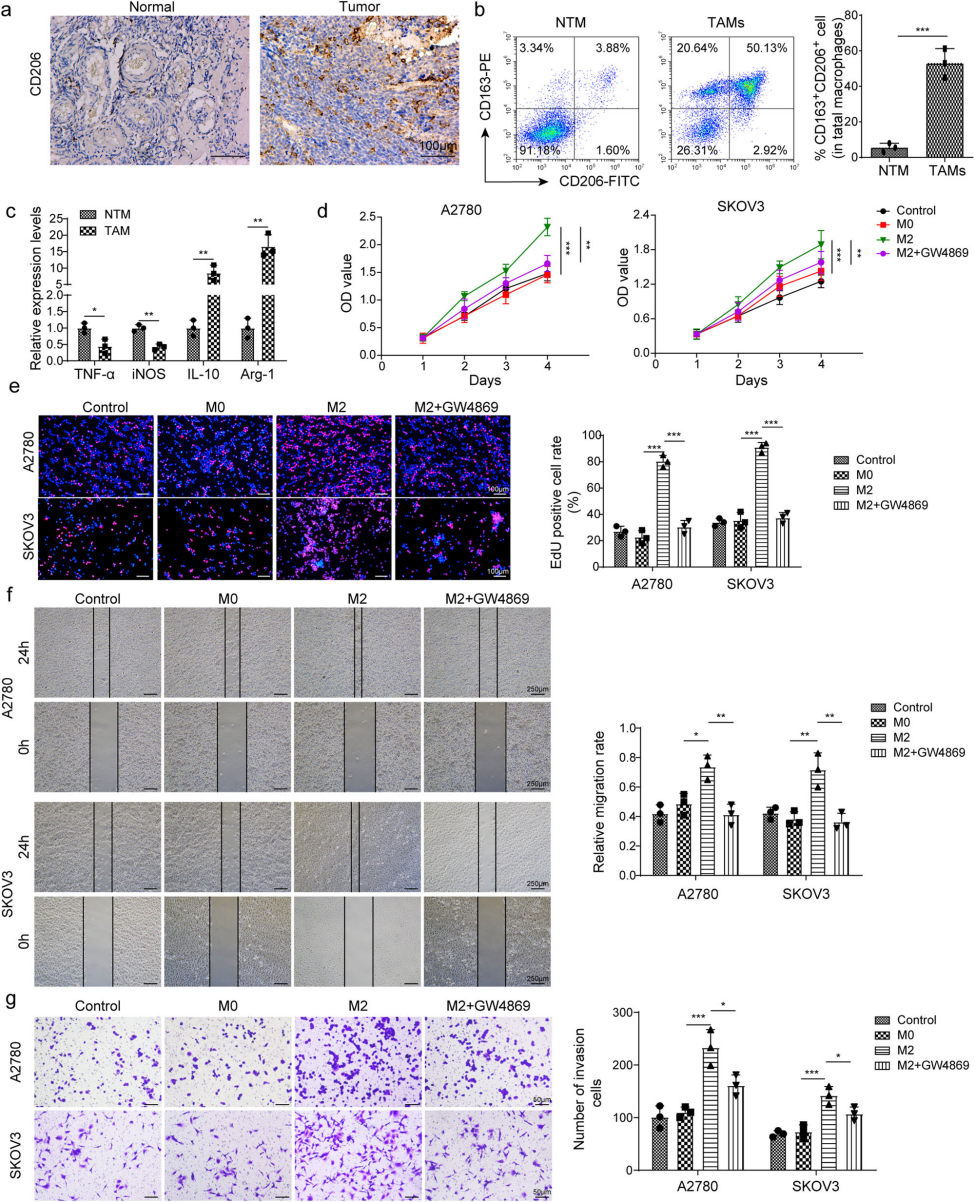
M2 macrophages were abundant in ovarian cancer and promoted malignant behaviors of cancer cells. **a** IHC staining of CD206 in ovarian cancer and normal tissues. Scale bar: 100 μm. **b** Flow cytometry analysis of CD163 and CD206-positive macrophages in NTMs and TAMs. **c** RT-qPCR analysis of *TNFα*, *iNOS*, *IL-10* and *Arg-1* in NTMs and TAMs. A2780 and SKOV3 cells were cultured with M0 or M2 macrophages. GW4869 was used to block exosome generation. **d**, **e** Cell proliferation was examined via CCK-8 and EdU incorporation assays (*n* = 3). Scale bar: 100 μm. **f**, **g** Migration and invasion were determined via wound healing and transwell assays (*n* = 3). Scale bar: 250 μm or 50 μm. *P < 0.05, **P < 0.01 and ***P < 0.001. Data were presented as mean ± standard deviation.

To investigate macrophage-mediated regulation of tumor progression, ovarian cancer tissues from patients were collected, and the M2 macrophage marker CD206 was detected via immunohistochemistry (IHC) staining. Compared to normal tissues, ovarian cancer tissues showed increased CD206-positive cells, suggesting increased abundance of M2 macrophages in ovarian cancer tissues (Fig. 1a). NTMs and TAMs were isolated, and the ratio of M2 macrophages (CD163 and CD206 double positive) was significantly increased in TAMs (Fig. 1b). Macrophages were isolated from tumor (TAM) and normal (NTM) tissues. Compared to NTMs, TAMs exhibited decreased the expression of M1 macrophage-related factors *TNF-α* and *iNOS* but increased the expression of M2 macrophage-associated factors *Arg-1* and *IL-10* (Fig. 1c). These data demonstrated that M2 macrophages were highly abundant in ovarian cancer. To explore whether M2 macrophages regulate the malignant behaviors of ovarian cancer cells, THP-1 cells were polarized to M2 macrophages, and A2780 and SKOV3 cells were cocultured with M0 or M2 macrophages. GW4869 was added to inhibit exosome generation. GW4869 significantly reduced total exosomal protein from M2 macrophages (Supplementary Fig. 1). Coculture with M2 macrophages greatly enhanced tumor cell proliferation, but it was abrogated by GW4869 (Fig. 1d, e). Furthermore, cell migration and invasion were enhanced by M2 macrophages, but these effects were largely suppressed by GW4869 treatment (Fig. 1f, g). These results suggest that abundant M2 macrophages promote malignancy in ovarian cancer cells via secreting exosomes.

### *CircTMCO3* is upregulated in ovarian cancer and M2 macrophage-derived exosomes can be internalized by ovarian cancer cells

**Fig. 2:**
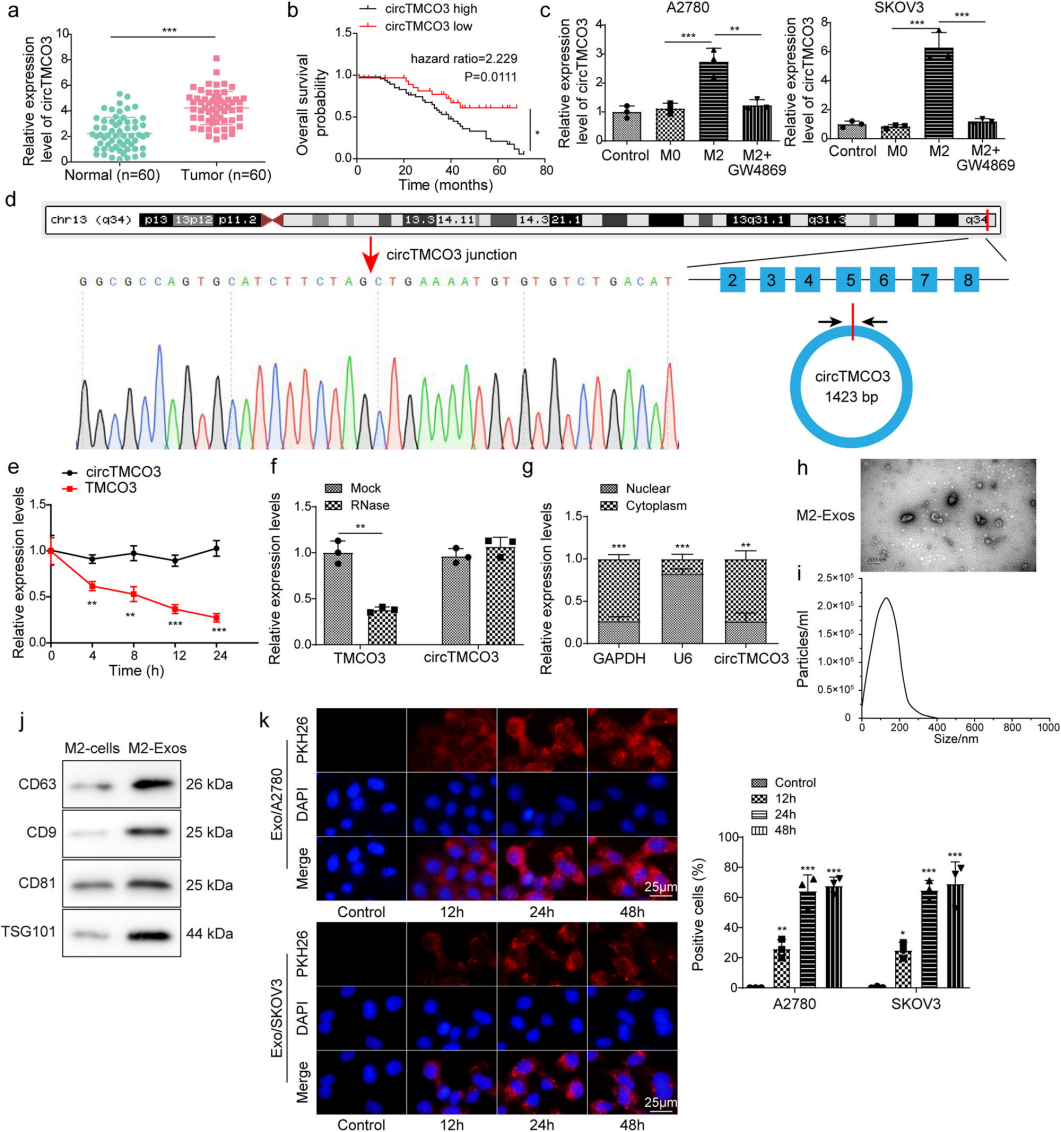
CircTMCO3 was upregulated and M2 macrophage-derived exosomes could enter ovarian cancer cells. **a** *CircTMCO3* expression was determined by RT-qPCR in patient tissues. **b** Patient survival. **c** RT-qPCR analysis of *circTMCO3* in A2780 and SKOV3 cells in coculture assays (*n* = 3). **d** Genomic loci and the junction site of *circTMCO3*. Sanger sequencing was applied to confirm the junction site. **e**, **f** The abundance of *circTMCO3* and linear *TMCO3* mRNA in SKOV3 in response to actinomycin D and RNase R treatment was determined through RT-qPCR (*n* = 3). **g** The abundance of *circTMCO3*, *U6* and *GAPDH* in nuclear and cytoplasmic fractions from SKOV3 cells was examined by RT-qPCR (*n* = 3). **h** The examination of M2 macrophage-derived exosomes using TEM. Scale bar: 200 nm. **i** Exosome size was evaluated by NTA. **j** Protein levels of CD63, CD9, CD81 and TSG101 in M2 macrophages and exosomes were analyzed by western blotting. **k** Exosomes (Red) were labeled with PKH26 and endocytosed by A2780 and SKOV3 cells (Nuclei, blue) after 12, 24 and 48 h. Scale bar: 25 μm. *P < 0.05, **P < 0.01 and ***P < 0.001. Data were presented as mean ± standard deviation.

We isolated exosomes from TAMs and NTMs and examined the expression of *hsa_circ_0000772*, *hsa_circ_0084335*, *hsa_circ_0031017* (*circTMCO3*), *hsa_circ_0062531*, *hsa_circ_0017992*, *hsa_circ_0091577*, and *hsa_circ_0065492* in TAMs, NTMs and exosomes. *hsa_circ_0000772*, *hsa_circ_0084335*, *hsa_circ_0031017* (*circTMCO3*) and *hsa_circ_0091577* were upregulated in both TAMs and exosomes from TAMs, and *hsa_circ_0031017* showed highest expression in TAMs (Supplementary Fig. 2a). Moreover, we examined the expression of *circTMCO3* in THP-1 cells, M0 and M2 macrophages and found that *circTMCO3* was highly expressed in M2 macrophages derived from THP-1 cells (Supplementary Fig. 2b). In addition, Northern blot assays showed that, compared to exosomes derived from NTMs, THP-1 cells and M0 macrophages, exosomes from TAMs and M2 macrophages derived from THP-1 cells showed increased *circTMCO3* expression, respectively (Supplementary Fig. 2c). As *circTMCO3* was abundant in cancer and TAM-derived exosomes, we analyzed *circTMCO3* expression in ovarian cancer tissues from patients. *CircTMCO3* expression was increased, and patients with high *circTMCO3* expression exhibited poor survival (Fig. 2a, b). Strikingly, coculture with M2 macrophages upregulated *circTMCO3* in A2780 and SKOV3 cells, and GW4869 treatment blocked *circTMCO3* upregulation (Fig. 2c), indicating that M2 macrophages might enhance *circTMCO3* expression through exosomes. As illustrated in Fig. 2d, *circTMCO3* is generated from the exons 2–8 of *TMCO3* on chromosome 13 with a length of 1423 nucleotides (nt) through back-splicing, and the junction site was verified by Sanger sequencing. Circular RNAs are quite stable in response to actinomycin D and RNase R treatment^24,25^. We found that, compared to linear *TMCO3* mRNA, *circTMCO3* exhibited decreased RNA decay after actinomycin treatment and high resistance to RNase R digestion (Fig. 2e, f). In addition, nuclear and cytoplastic fractions were prepared, and *circTMCO3* could be detected in both nuclear and cytoplastic fractions, but it primarily localized in the cytoplasm (Fig. 2g). GW4869 suppressed *circTMCO3* expression and M2-meditated oncogenic activity, suggesting that exosomes generated by M2 macrophages might be implicated in regulating *circTMCO3* expression and ovarian cancer progression. Exosomes were then examined by transmission electron microscopy (TEM) and nanoparticle tracking analysis (NTA). Distinctive round bilayer vesicles with mainly 30–150 nm in diameter were observed (Fig. 2h, i). Moreover, M2 macrophage-derived exosomes showed high levels of extracellular vesicle proteins including tetraspanins (CD63, CD9, CD81) and TSG101^26^ compared to M2 macrophages (Fig. 2j). In coculture assays, we found that PKH26-labeled exosomes were internalized by ovarian cancer cells (Fig. 2k). The ratio of PKH26-positive cells was ~30% at 12 h, and it was increased to ~65% at 24 and 48 h (Fig. 2k). These results suggest that M2 macrophages raise *circTMCO3* level in cancer cells via exosomes.

### M2 macrophage-derived exosomes promote the malignancy by delivering *circTMCO3*

**Fig. 3:**
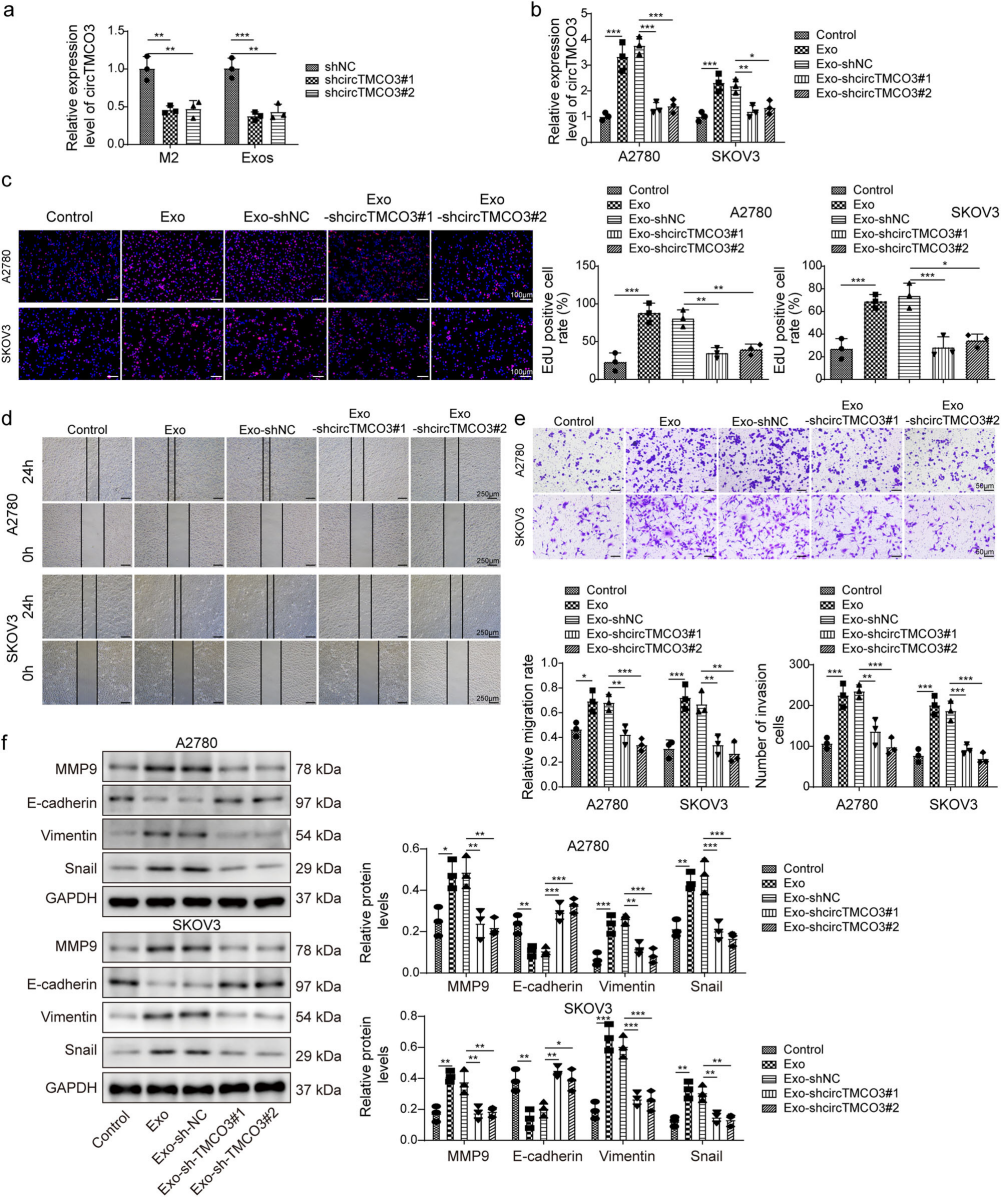
M2-derived exosomes promoted the malignancy of ovarian cancer cells by delivering circTMCO3. **a** RT-qPCR analysis of *circTMCO3* in M2 macrophages transfected with shNC, *shcircTMCO3*#1 or #2 (*n* = 3). A2780 and SKOV3 cells were cocultured with Exo, Exo-shNC, Exo-shcircTMCO3#1 or Exo-shcircTMCO3#2. **b** RT-qPCR analysis of *circTMCO3* (*n* = 3). **c** EdU (red) incorporation analysis (*n* = 3; Scale bar, 100 µm; Nuclei, blue). **d** Wound healing assays for cell migration analysis (*n* = 3). Scale bar: 250 μm. **e** Transwell assays for cell invasion analysis (*n* = 3). Scale bar: 50 μm. **f** Protein levels of MMP9, E-cadherin, Vimentin and Snail were detected via western blotting. *P < 0.05, **P < 0.01 and ***P < 0.001. Data were presented as mean ± standard deviation.

We proposed that M2 macrophage-derived exosomes might exert functions by delivering *circTMCO3*. Thus, *circTMCO3* expression was knocked down by transfection of *shcircTMCO3*#1 or #2 in M2 macrophages, and M2-derived exosomes showed decreased *circTMCO3* expression (Fig. 3a). A2780 and SKOV3 cells cocultured with Exo or Exo-shNC showed elevated expression of *circTMCO3*, but Exo-shcircTMCO3#1 and #2 had no significant effect on the expression of *circTMCO3* in ovarian cancer cells (Fig. 3b), suggesting that M2 macrophage-derived exosomes delivered *circTMCO3* into ovarian cancer cells. We found that Exo or Exo-shNC significantly raised 5-ethynyl-2′-deoxyuridine (EdU)-positive cell rate, migration, and invasion (Fig. 3c–e). However, Exo-shcircTMCO3#1 and #2 had no significant effect on the proliferation, migration, and invasion of ovarian cancer cells (Fig. 3c–e). Furthermore, compared to controls, A2780 and SKOV3 cells cocultured with Exo or Exo-shNC exhibited increased the expression of MMP9, Vimentin and Snail but decreased the expression of E-cadherin that were highly associated with cancer invasiveness and metastasis^27–30^, but these effects were abolished by Exo-shcircTMCO3#1 or #2 coculture (Fig. 3f). Collectively, these observations indicate that M2 macrophage-derived exosomal *circTMCO3* enhances ovarian cancer cell proliferation, migration, and invasion. We further evaluated whether *shcircTMCO3*#1 and #2 reprogramed macrophages. Compared to Control, M2 and *shNC*, *shcircTMCO3*#1 and #2 significantly reduced CD163-positive M2 macrophages (Supplementary Fig. 3a). Moreover, *shcircTMCO3*#1 and #2 repressed the expression of M2-related markers including IL-10, Arg-1, Fizz-1 and TGF-β in M2 macrophages (Supplementary Fig. 3b–f), suggesting that inhibition of *circTMCO3* suppressed M2 macrophage polarization.

### Exosomal *circTMCO3* functions as a ceRNA for *miR-515-5p* in ovarian cancer

**Fig. 4:**
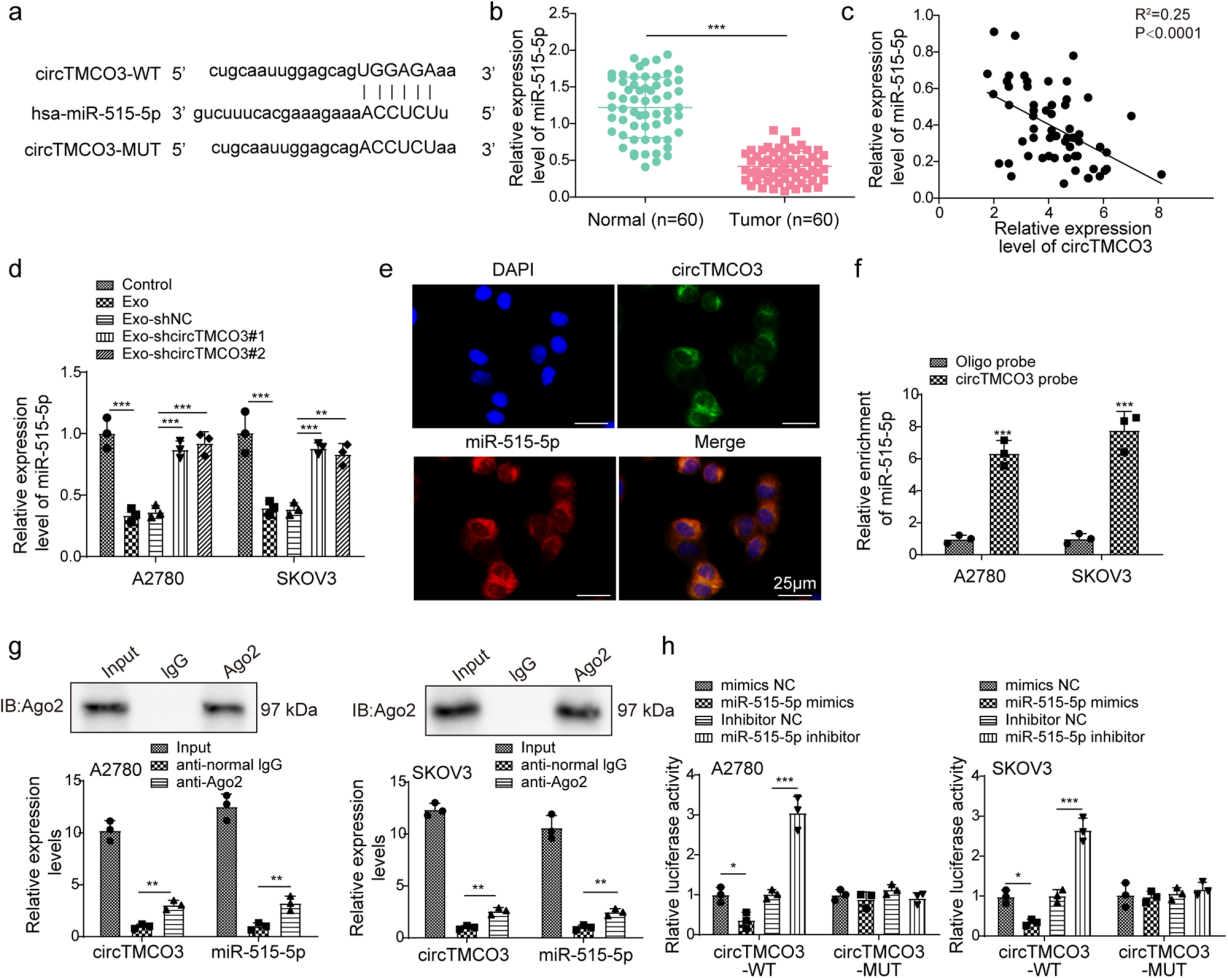
Exosomal circTMCO3 functioned as a ceRNA for miR-515-5p in ovarian cancer. **a** The *hsa-miR-515-5p* binding site in *circTMCO3*. **b** RT-qPCR analysis of *miR-515-5p* in normal and tumor groups. **c** Pearson correlation analysis of *circTMCO3* and *miR-515-5p* in tumor group. **d** RT-qPCR analysis of *miR-515-5p* in A2780 and SKOV3 cells in coculture assays (*n* = 3). **e** The colocalization of *circTMCO3* and *miR-515-5p* were determined with FISH. Cells were hybridized with Alexa Fluor 488-labeled *circTMCO3* (Green) and Cy3-labeled *miR-515-5p* (Red) probes, and the nuclei were stained with DAPI (Blue). Scale bar: 25 μm. **f** Direct binding of *circTMCO3* and *miR-515-5p* was determined by RNA pull-down assays (*n* = 3). **g** The interaction of *circTMCO3* and *miR-515-5p* was determined by RIP assays (*n* = 3). **h** Luciferase activity of wildtype (circTMCO3-WT) or mutant (circTMCO3-MUT) *circTMCO3* reporters in A2780 and SKOV3 cells transfected with *miR-515-5p* mimics or inhibitor (*n* = 3). *P < 0.05, **P < 0.01 and ***P < 0.001. Data were presented as mean ± standard deviation.

As circRNAs regulate the expression of target genes via sponging miRNAs, a potential *miR-515-5p* binding site in *circTMCO3* was predicted through Circinteractome (Fig. 4a). Ovarian cancer tissues showed low *miR-515-5p* expression, and *miR-515-5p* expression was negatively correlated with *circTMCO3* expression (Fig. 4b, c). Besides, *miR-515-5p* was downregulated by Exo and Exo-shNC in A2780 and SKOV3 cells, but Exo-shcircTMCO3#1 and #2 lost the suppressive capacity (Fig. 4d). FISH assays showed colocalization of *circTMCO3* and *miR-515-5p* in the cytoplasm (Fig. 4e). Furthermore, we found that *miR-515-5p* could be efficiently enriched by the *circTMCO3* probe (Fig. 4f), and both *circTMCO3* and *miR-515-5p* were enriched by an anti-Ago2 (Fig. 4g). The luciferase activity of *circTMCO3*-WT was inhibited by *miR-515-5p* overexpression but reinforced by *miR-515-5p* knockdown in ovarian cancer cells (Fig. 4h). However, neither overexpression nor knockdown of *miR-515-5p* affected the luciferase activity of *circTMCO3*-MUT (Fig. 4h). These data demonstrate that *circTMCO3* acts as a *miR-515-5p* sponge to reduce its expression in ovarian cancer.

### *miR-515-5p* directly targets *ITGA8* to inhibit its expression in ovarian cancer

**Fig. 5:**
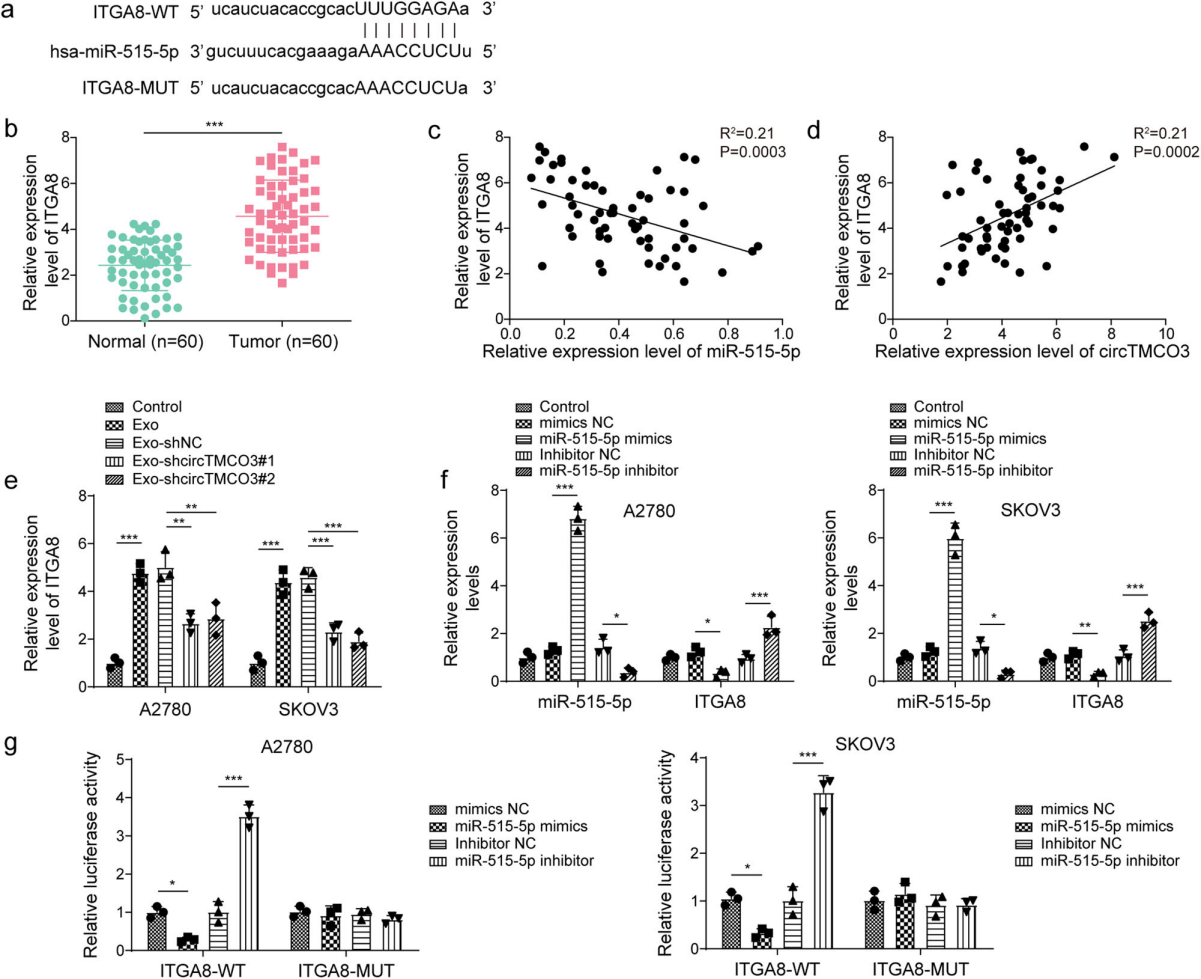
miR-515-5p directly targeted ITGA8 in ovarian cancer. **a** The binding site for *hsa-miR- 515-5p* in the 3′ UTR of *ITGA8*. **b** RT-qPCR analysis of *ITGA8* in patient tissues. **c**, **d** Pearson correlation analysis of *ITGA8* and *miR-515-5p* or *circTMCO3* in the tumor group. **e** RT-qPCR analysis of *ITGA8* in A2780 and SKOV3 cells in coculture assays (*n* = 3). **f** The expression of *miR-515-5p* and *ITGA8* in A2780 and SKOV3 cells transfected with *miR-515-5p* mimics, inhibitor, or negative controls (*mimics* and *inhibitor NC*) was determined via RT-qPCR (*n* = 3). **g** Luciferase activity of wildtype (ITGA8-WT) or mutant (ITGA8-MUT) ITGA8 reporters (*n* = 3). *P < 0.05, **P < 0.01 and ***P < 0.001. Data were presented as mean ± standard deviation.

To further explore downstream targets of *miR-515-5p*, we predicted a potential binding site for *miR-515-5p* in the 3′ untranslated region (UTR) of *ITGA8* (Fig. 5a). Tumor tissues showed high expression of *ITGA8* compared to normal tissues (Fig. 5b). *ITGA8* expression was negatively correlated with *miR-515-5p* expression but positively correlated with *circTMCO3* expression in ovarian cancer (Fig. 5c, d). Moreover, Exo and Exo-shNC significantly promoted *ITGA8* expression, but it was abolished by knockdown of *circTMCO3* (Fig. 5e). *miR-515-5p* was overexpressed or knocked down in A2780 and SKOV3 cells (Fig. 5f). *ITGA8* was markedly reduced by *miR-515-5p* overexpression but elevated by *miR-515-5p* silencing (Fig. 5f). *miR-515-5p* overexpression impaired the luciferase activity of ITGA8-WT, but *miR-515-5p* knockdown raised its luciferase activity (Fig. 5g). The luciferase activity of ITGA8-MUT was not affected by transfection of *miR-515-5p* mimics or inhibitor (Fig. 5g). Therefore, *miR-515-5p* represses ITGA8 expression via binding to its 3′ UTR in ovarian cancer.

### *miR-515-5p* attenuates malignancy in ovarian cancer via downregulating ITGA8

**Fig. 6:**
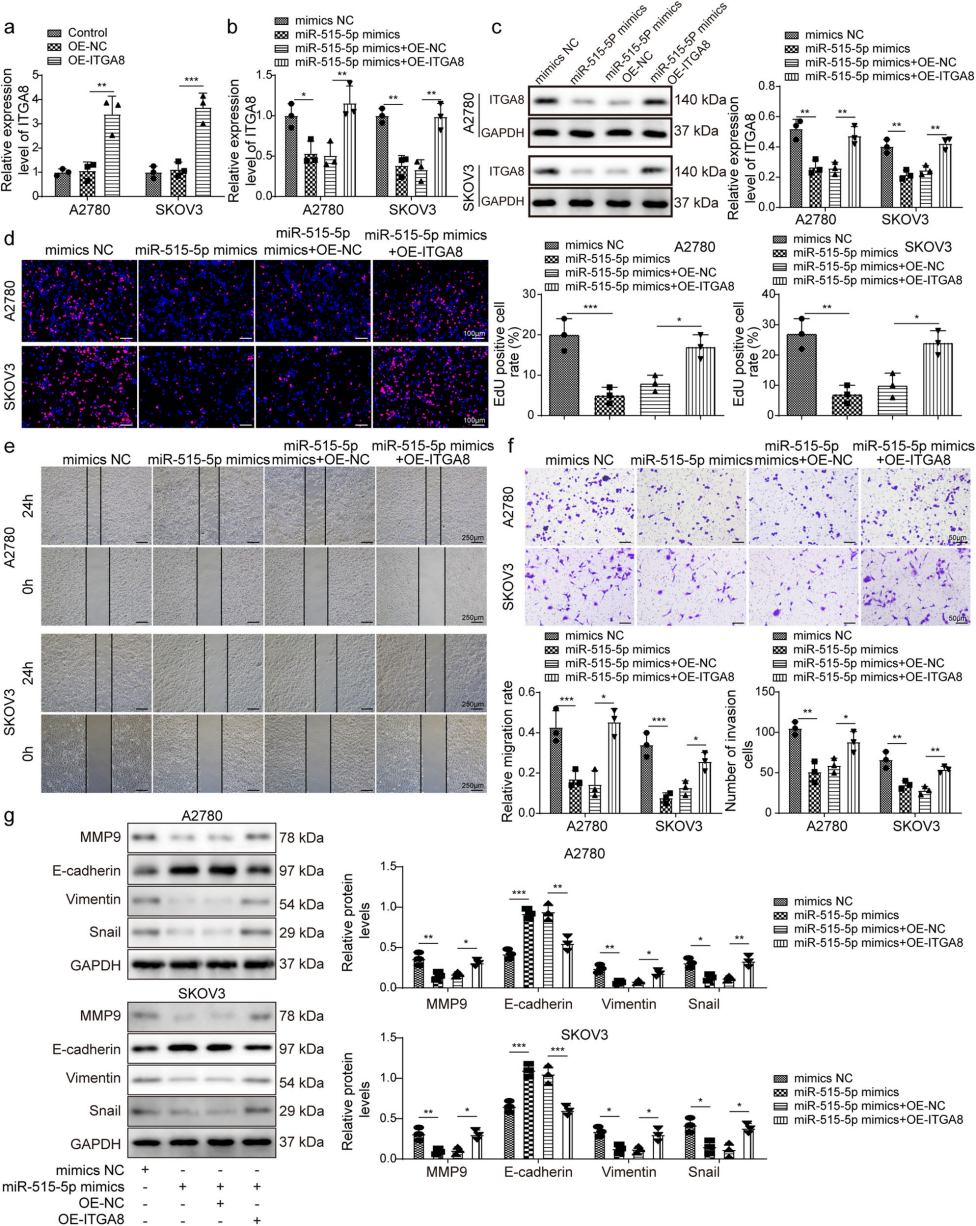
miR-515-5p attenuated malignancy in ovarian cancer via downregulating ITGA8. **a** RT-qPCR analysis of *ITGA8* in A2780 and SKOV3 cells transfected with OE-NC and OE-ITGA8 (*n* = 3). A2780 and SKOV3 cells were transfected with *mimics NC*, *miR-515-5p mimics*, *miR-515-5p mimics* in combination with OE-NC or OE-ITGA8. **b**, **c** RT-qPCR and western blotting analysis of *ITGA8* (*n* = 3). **d** EdU incorporation analysis (*n* = 3). Scale bar: 100 μm. **e** Wound healing assays (*n* = 3). Scale bar: 250 μm. **f** Transwell assays (*n* = 3). Scale bar: 50 μm. **g** Protein levels of MMP9, E-cadherin, Vimentin and Snail were detected through western blotting. *P < 0.05, **P < 0.01 and ***P < 0.001. Data were presented as mean ± standard deviation.

To confirm whether *miR-515-5p* exerts anti-tumoral effects through ITGA8, ITGA8 was overexpressed in A2780 and SKOV3 cells through OE-ITGA8 transfection (Fig. 6a). Moreover, *miR-515-5p* mimics-mediated downregulation of *ITGA8* was reversed by simultaneous overexpression of ITGA8 (Fig. 6b, c). *miR-515-5p* overexpression reduced EdU-positive cell rate and inhibited migration and invasion, but these suppressive effects were abrogated by overexpression of ITGA8 (Fig. 6d–f). The expression of MMP9, Vimentin and Snail were downregulated, and E-cadherin was upregulated in *miR-515-5p*-overexpressing cells, but simultaneous overexpression of ITGA8 reversed their expression (Fig. 6g). To conclude, *miR-515-5p* represses proliferation, migration, invasion, and epithelial-mesenchymal transition (EMT) through suppression of ITGA8 in ovarian cancer.

### Exosomal *circTMCO3* promotes malignancy of ovarian cancer cells through the *miR-515-5p/ITGA8* axis

**Fig. 7:**
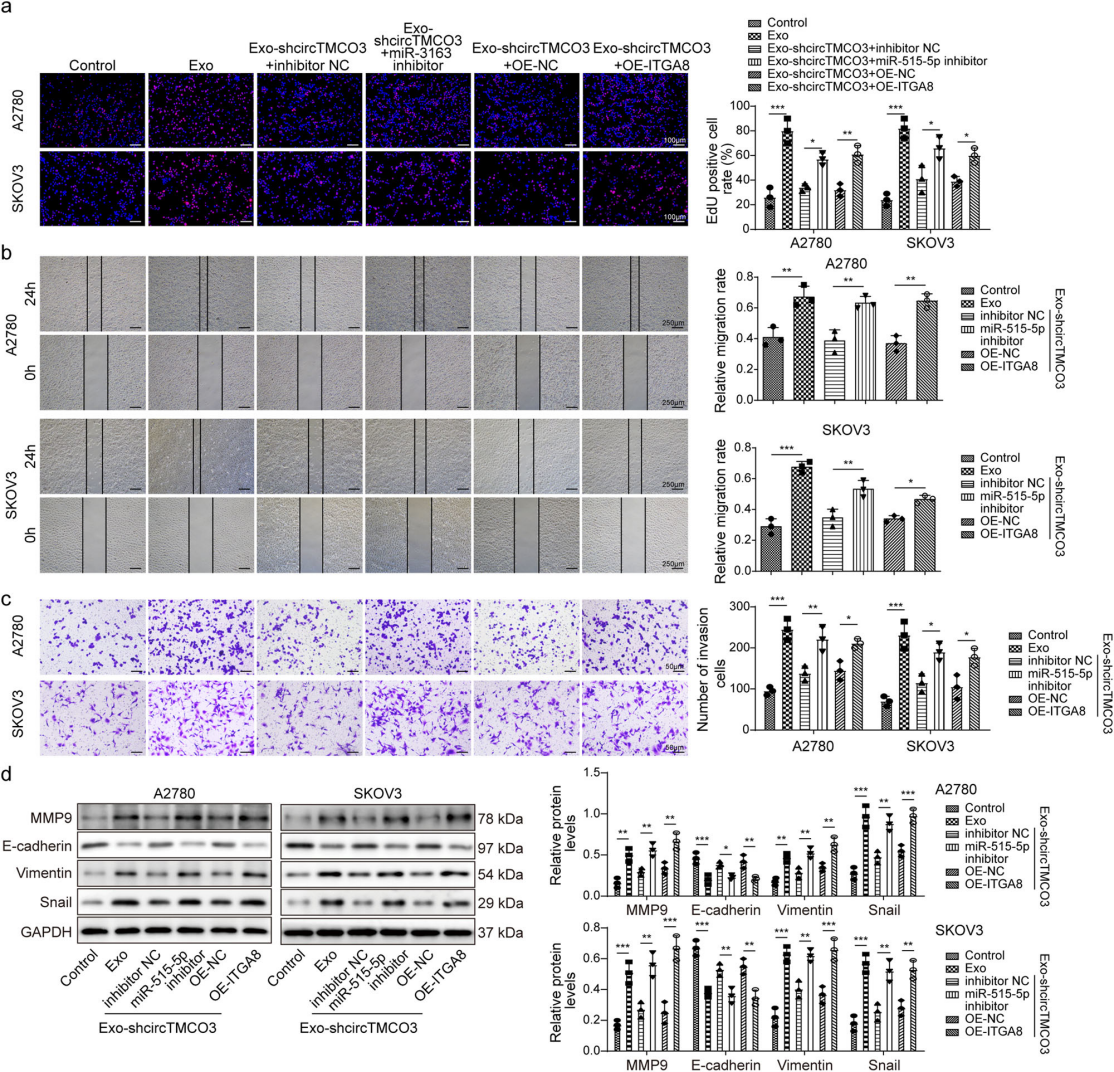
Exosomal circTMCO3 promoted malignancy of ovarian cancer cells through the miR-515-5p/ITGA8 axis. A2780 and SKOV3 cells were transfected with *miR-515-5p* inhibitor or OE-ITGA8 and cocultured with Exo-shcircTMCO3#1 or Exo-shcircTMCO3#2. **a** EdU incorporation analysis (*n* = 3). Scale bar: 100 μm. **b** Wound healing assays (*n* = 3). Scale bar: 250 μm. **c** Transwell assays (*n* = 3). Scale bar: 50 μm. **d** Protein levels of MMP9, E-cadherin, Vimentin and Snail were detected via western blotting. *P < 0.05, **P < 0.01 and ***P < 0.001. Data were presented as mean ± standard deviation.

We then examined whether exosomal *circTMCO3*-mediated regulation of malignancy in ovarian cancer was dependent on the *miR-515-5p/ITGA8* axis. Exo-mediated acceleration of proliferation, migration, and invasion was suppressed by knockdown of *circTMCO3* (Fig. 7a–c). Simultaneous knockdown of *miR-515-5p* or overexpression of ITGA8 reversed these effects and promoted proliferation, migration, and invasion (Fig. 7a–c). The expression of MMP9, Vimentin and Snail was enhanced, and E-cadherin was downregulated by Exo, but not by Exo-shcircTMCO3#1 and #2 (Fig. 7d). Knockdown of *miR-515-5p* or overexpression of ITGA8 reversed Exo-shcircTMCO3-mediated effects on these gene expressions (Fig. 7d). Therefore, exosomal *circTMCO3* strengthens malignancy via targeting the *miR-515-5p/ITGA8* axis.

### M2 macrophage-derived exosomal *circTMCO3* accelerates ovarian cancer progression in vivo

**Fig. 8:**
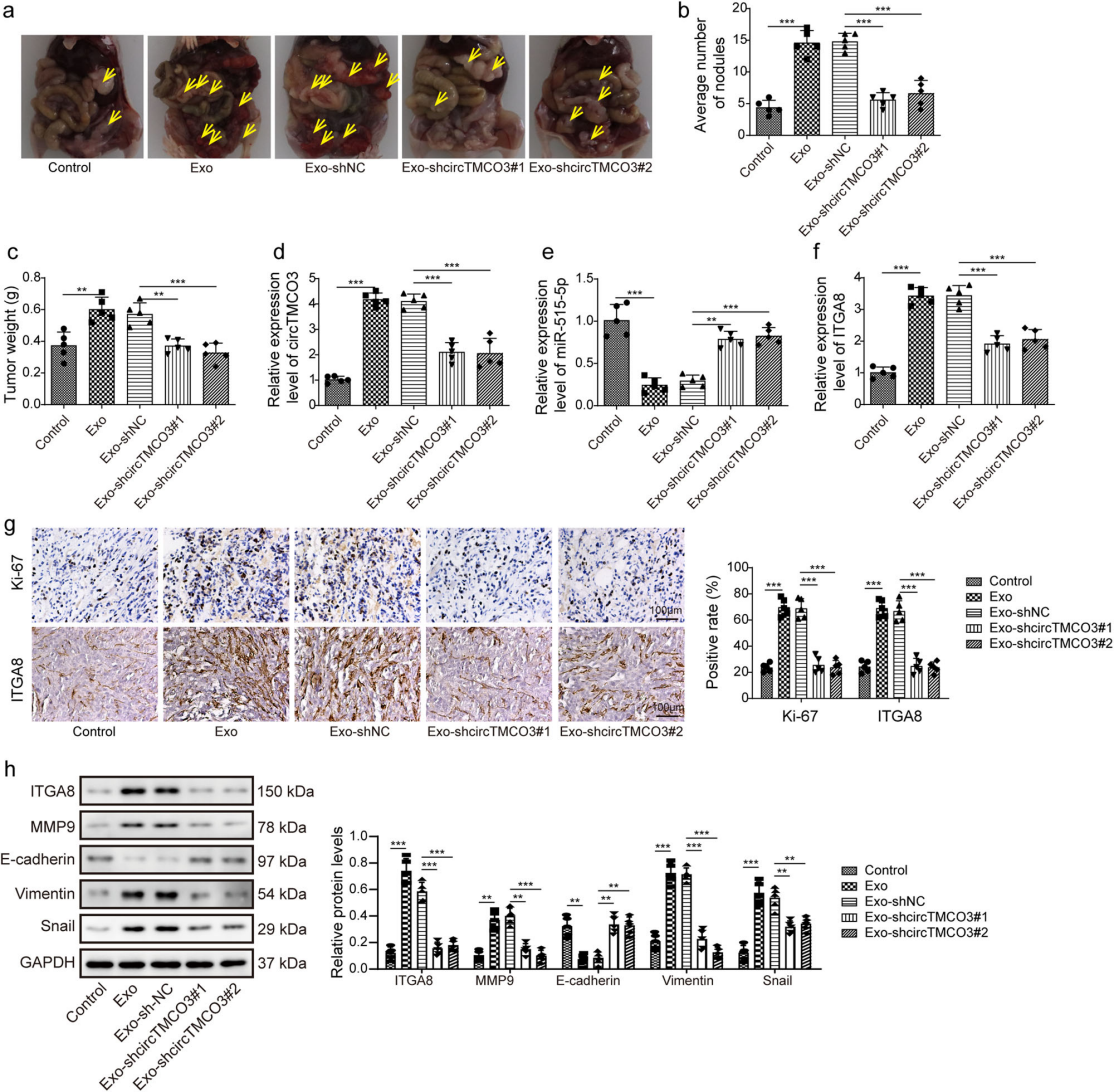
M2 macrophage-derived exosomal circTMCO3 accelerated ovarian cancer progression. Mice were intraperitoneally injected with 5 × 10^6^ SKOV3 cells and administrated with control (PBS), Exo, Exo-shNC, Exo-shcircTMCO3#1 or Exo-shcircTMCO3#2. **a** Mouse abdomen was opened and imaged. **b** Tumor nodules were counted (*n* = 5). **c** Tumor weight (*n* = 5). **d**–**f** RT-qPCR analysis of *circTMCO3*, *miR-515-5p* and *ITGA8* in tumors (*n* = 5). **g** IHC staining of Ki67 and ITGA8 in tumors. Scale bar: 100 μm. **h** Protein levels of ITGA8, MMP9, E-cadherin, Vimentin and Snail in tumors were examined by Western blotting. **P < 0.01 and ***P < 0.001. Data were presented as mean ± standard deviation.

We established an ovarian cancer xenograft mouse model via intraperitoneal injection, and mice were administrated with Exo, Exo-shNC, Exo-shcircTMCO3#1 or Exo-shcircTMCO3#2. Compared to control, Exo and Exo-shNC administration promoted tumor growth and raised tumor nodules and weight (Fig. 8a–c). However, Exo-shcircTMCO3#1 and #2 failed to exert these oncogenic effects (Fig. 8a–c). Moreover, we observed that Exo and Exo-shNC promoted the expression of *circTMCO3* and *ITGA8* and inhibited *miR-515-5p* expression, but it was abrogated by *circTMCO3* knockdown (Fig. 8d–f). IHC staining showed that Exo and Exo-shNC administration enhanced the expression of Ki67, a widely used proliferation marker of cancer cells, and ITGA8 in tumors, and knockdown of *circTMCO3* abrogated exosome-mediated upregulation of Ki67 and ITGA8 (Fig. 8g). Furthermore, ITGA8, MMP9, Vimentin and Snail were upregulated, and E-cadherin was downregulated by Exo and Exo-shNC administration, and these effects were suppressed by Exo-shcircTMCO3#1 and #2 (Fig. 8h). Collectively, these results demonstrate that M2 macrophage-derived exosomal *circTMCO3* accelerates ovarian cancer progression in vivo.

## Discussion

Ovarian cancer is a highly lethal cancer that accounts for 5% of all cancer-related deaths among women^31,32^. Ovarian cancer is generally diagnosed at a late stage, causing poor outcome of patients^33^. Therefore, exploring the regulatory mechanisms of ovarian cancer progression is very important. Here, we firstly demonstrated that M2 macrophage-derived exosomal *circTMCO3* sponged *miR-515-5p* and relieved its inhibitor effect on ITGA8 expression, thereby promoting the malignancy and progression of ovarian cancer.

Exosomes play vital roles in cell-cell communication via transferring cell components and regulating behaviors of various cancer cells^34,35^. M2 macrophages attract much attention as the major component of tumor microenvironment. Exosomes released by M2 macrophages promote the metastasis of hepatocellular carcinoma cells and accelerate angiogenesis and growth of pancreatic cancer^36,37^. In consistence, we observed that M2 macrophages enhanced ovarian cancer cell proliferation, migration, and invasion via releasing exosomes. Exosomes have shown enormous potential in drug delivery with great biocompatibility and low immunogenicity^38^. Our data suggests that M2 macrophage-derived exosomes can be engineered for the management of ovarian cancer.

In recent years, exosomes delivering ncRNAs, such as lncRNAs, are becoming a pivotal regulator in regulating cancer progression^39^. Exosomal circRNAs have been regarded as potential therapeutic targets for various cancers^40^. Ma et al. found that exosomal *circRNA051239* derived from tumors promoted proliferation, migration, and invasion of epithelial ovarian cancer cells via working as a *miR-509-5p* sponge and regulating PRSS3 expression^41^. In addition, circRNAs are key regulators of ovarian cancer progression and therapeutic targets for ovarian cancer^42^. However, our understanding of circRNAs and related regulatory mechanisms are still limited. *CircTMCO3* exerts an oncogenic activity in gastric cancer, but its roles in other cancers including ovarian cancer remain unknown. Here, increased expression of *circTMCO3* in M2 macrophages suggested us that exosomes might carry *circTMCO3* to exert its function in ovarian cancer. Indeed, exosomes with knockdown of *circTMCO3* lost their activity in regulating malignancy, demonstrating that exosomes exert their function via delivering *circTMCO3* in ovarian cancer.

The ceRNA hypothesis suggests that lncRNAs work as miRNA sponges to upregulate downstream mRNA targets in cancers^43^. *miR-577* has been identified as a target of *circTMCO3*^18^. Wen et al. discovered that *miR-515-5p* repressed malignant behaviors in breast cancer through *CBX4*^44^. *miR-515-5p* restrained migration and metastasis by downregulating MARK4 in lung and breast cancers^45^. However, the implication of *miR-515-5p* in ovarian cancer is unknown. Here, *miR-515-5p* was identified as a target of *circTMCO3*, and *miR-515-5p* restrained proliferation, migration, invasion, and EMT, discovering an anti-tumor activity in ovarian cancer.

The integrin family serves key roles in promoting cancer progression via facilitating proliferation, migration, invasion, and metastasis in various cancers^46^. As an integrin subunit, ITGA8 promotes EMT, migration, and invasion in multiple myeloma^23^, and its diagnostic and prognostic values in colon cancer have been validated^47^. However, its roles in ovarian cancer have never been reported previously. We showed *ITGA8* as a downstream target of *miR-515-5p* in ovarian cancer. Our findings suggested that *miR-515-5p* reduced ITGA8 expression via binding to its 3′ UTR in ovarian cancer, and ITGA8 overexpression reversed *miR-515-5p*-mediated regulation of cancer malignancy and progression, indicating that *miR-515-5p* reduced ITGA8 expression to suppress proliferation, migration, invasion, and metastasis. However, ITGA8 is transmembrane receptor that mediates numerous cellular processes, and its implication in exosome internalization needs to be explored. Besides regulating ITGA8 expression, exosomal RNA-mediated regulation of ITGA8 function might be implicated in the pathogenesis of ovarian cancer.

Taken together, M2 macrophage-derived exosomes enhance malignancy in ovarian cancer by delivering *circTMCO3* and targeting the *miR-515-5p/ITGA8* axis. Our findings highlight exosomal *circTMCO3*-mediated regulation of malignancy of cancer cells and provide potential therapeutic strategies for ovarian cancer. Single cell sequencing will be performed in future studies to investigate the gene expression level difference between the cell types within the tumor and normal tissues.

## Methods

### Patients and specimens

Sixty patients were diagnosed with ovarian cancer at Hunan Cancer Hospital, and tumor and adjacent normal tissues were collected and stored for RNA extraction and immunohistochemistry staining. Inclusion criteria was following: 1 Greater than or equal to 18 years old; 2 First diagnosis with primary ovarian cancer; 3 No other severe diseases, such as cancers and diabetes; 4 No treatment; 5 No pregnancy and lactation. Macrophages were isolated from tumor (TAM) and normal (NTM) tissues and stained with CD11b and CD206 antibodies (101205 and 321105, Biolegend, San Diego, CA, USA). The cells were detected Beckman cytoflex flow cytometry (Beckman, USA), and the Cytexpert Software was applied for the flow cytometry data analysis. The gating strategy was provided in the Supplementary Fig. 4. Written informed consent was required from patients. The Ethics Committee of Hunan Cancer Hospital approved this study.

### Cell culture and polarization

Human ovarian cancer cells, A2780 and SKOV3, were purchased from iCell Bioscience Inc (Shanghai, China) and Cell Bank, Chinese Academy of Sciences (Shanghai, China) and maintained in DMEM/10% FBS (Gibco, Waltham, MA, USA). Human monocytic cell THP-1 was bought from the American Type Culture Collection (ATCC, Manassas, VA, USA). Cells were authenticated by STR profiling and tested for mycoplasma contamination. For macrophage polarization, THP-1 cells were induced with PMA (100 ng/mL, Sigma, St. Louis, MO, USA) for 48 h (M0) prior to additional 24-h incubation with LPS (100 ng/mL, Sigma) and IL-4 (20 ng/mL, PeproTech, Cranbury, NJ, USA) for M2 polarization. For 24-h coculture of macrophages and ovarian cancer cells, M0 or M2 macrophages (1 × 10^5^) were plated on the upper chambers, and A2780 or SKOV3 cells (5 × 10^5^) were seeded on the lower chambers. For GW4869 treatment, M2 macrophages were pretreated with GW4869 (10 μM, Sigma) for 8 h.

### Cell transfection

For ITGA8 overexpression, the coding region of *ITGA8* was cloned into pcDNA3.1 (OE-ITGA8, Invitrogen, Carlsbad, CA, USA). Mock transfection controls were prepared using the empty pcDNA3.1 vector (OE-NC). The shRNAs against *circTMCO3* (*shcircTMCO3*#1 and #2), *miR-515-5p* mimics, inhibitor, and negative controls (*shNC*, *mimics NC* and *inhibitor NC*) were synthesized by RiboBio (Guangzhou, China). A2780 and SKOV3 cells were transfected using Lipo 3000 (Invitrogen) following the manual. For knockdown of *circTMCO3*, lentiviral particles were prepared and transduced into M2 macrophages. After 48 h, cells were harvested. The sequences of *shcircTMCO3*#1 and #2 were 5′-GTGCATCTTCTAGCTGAAAAT-3′ and 5′-GCATCTTCTAGCTGAAAATGT-3′.

### Exosome extraction, characterization, and internalization

Exosomes were extracted from M2 macrophages using Total Exosome Isolation Reagent (Invitrogen). Culture media were centrifuged, and the supernatants were collected. The exosome isolation reagent was mixed with the supernatants thoroughly and incubated overnight at 4 °C. Samples were centrifuged, and the pellet was resuspended. Exosomes were quantified and co-cultured with A2780 or SKOV3 cells at 100 µg/mL for 48 h. TEM was applied to examine exosome morphology. exosome size was analyzed by NTA (Malvern, Westborough, MA, USA). Protein levels of CD9, CD63, CD81 and TSG101 were determined by western blotting. Exosomes were stained with PKH26 dye (Sigma) and incubated with A2780 and SKOV3 cells for 24 h for imaging.

### *CircTMCO3* characterization

SKOV3 cells were treated with actinomycin D (5 µg/mL, Sigma) for 0, 4, 8, 12, or 24 h, and RNA was extracted for analyzing the decay of *circTMCO3* and *TMCO3* mRNA. Moreover, RNA was isolated, treated with RNase R (5 U/µg, Abcam, Cambridge, UK) for 2 h and subjected RT-qPCR for examining the remaining of *circTMCO3* and *TMCO3* mRNA. *CircTMCO3* was amplified using divergent primers, and the junction site was identified through Sanger sequencing (Sangon Biotech, Shanghai, China).

### Nuclear and cytoplasmic fractionation

Nuclear/Cytosol Fractionation Kit was purchased from BioVision (Milpitas, CA, USA). Nuclear and cytoplasmic fractions were separated following the manual. The abundance of *circTMCO3*, *U6* snRNA and *GAPDH* in nuclear and cytoplasmic fractions was examined by RT-qPCR.

### Fluorescence in situ hybridization (FISH)

SKOV3 cells were seeded on coverslips and fixed in a mixture solution of methanol and acetic acid (3:1) for 10 min. Let coverslips dried naturally, and cells were immersed in hybridization solution containing Alexa Fluor 488-labeled *circTMCO3* and Cy3-labeled *miR-515-5p* probes at 25 nM. Coverslips were denatured and incubated overnight. Coverslips were washed and mounted in antifade mountant with DAPI (Beyotime, Shanghai, China) for imaging.

### Real-time quantitative reverse transcription-PCR (RT-qPCR)

RNA was isolated using Total RNA Isolation Kit (Thermo Fisher Scientific, Waltham, MA, USA) and reversely transcribed into cDNA. The mirPremier microRNA isolation kit (Sigma) was used to isolate miRNAs, which were reversely transcribed using the miScript kit (QIAGEN, Germantown, MD, USA). Quantitative PCR was applied to detect *circTMCO3*, *TMCO3*, *miR-515-5p*, *ITGA8*, *TNF-α*, *iNOS*, *IL-10*, *Arg-1* using SYBR Green (Beyotime). *GAPDH* and *U6* snRNA were used as normalization controls. The 2^−∆∆Ct^ method was used to calculate their relative expression. Primers were listed in Supplementary Table 1.

### Western blotting

Exosomal protein was isolated using Exosome RNA and Protein Isolation Kit (ThermoFisher Scientific). Tumor tissues were homogenized and A2780 and SKOV3 cells were suspended in Protein Extraction Buffer (Abcam) supplemented with protease inhibitors (Thermo Fisher Scientific), and the supernatants were collected after centrifugation. Protein concentration was determined with BCA Protein Assay Kit (Beyotime) according to the manufacturer’s suggestion. After quantification, protein was loaded at 30 µg each lane, electrophoresed, and transferred to polyvinylidene fluoride (PVDF) membranes (Bio-Rad, Hercules, CA, USA). The PVDF membranes were cropped according to the protein molecular weight recommended by the manufacturer’s instructions. Rabbit antibodies against CD9 (1:1000, ab223052), CD63 (1:2000, ab231975), CD81 (1:2000, ab155760), TSG101 (1:800, ab225877), MMP9 (1:500, ab283575), E-cadherin (1:1000, ab227639), Vimentin (1:500, ab137321), Sanil (1:500, ab216347), ITGA8 (1:1000, ab243027) and GAPDH (1:8000, ab9485) from Abcam (Cambridge, UK) were used to examine their protein levels. GAPDH was used a normalization control. An HRP-donkey anti-rabbit IgG antibody (A16023, ThermoFisher Scientific) was used to incubate membranes for 1 h. Protein bands were visualized using ECL substrate (Beyotime) and quantified with the ImageJ software. The original western blot images were presented Supplementary Fig. 5.

### Northern blotting

Cells and exosomes were lysed, and total RNA was extracted and quantified by measuring the OD at 260 nm. Subsequently, RNA was separated by agarose gel electrophoresis and transferred to Nylon membrane. RNA was cross-linked to the membrane, prehybridized, and hybridized with the radiolabeled *circTMCO3* probe. Then, the membrane was washed, and the signal was visualized using X-ray^27^.

### Cell Counting Kit-8 (CCK-8) assay

After cell treatment, culture medium was removed, and 100 µL of medium was added. CCK-8 (10 µL, Sigma) was added and incubated for 4 h. The absorbance (450 nm) was recorded.

### Wound healing assay

In brief, inserts were oriented in plates. A2780 and SKOV3 cells (5 × 10^5^) were added and cultured at 37 °C. Then, inserts were removed, and cells were washed and cocultured with macrophages or exosomes as indicated. The wound healing process was monitored using the BX51 microscope from Olympus (Tokyo, Japan).

### EdU incorporation assay

A2780 and SKOV3 cells were cocultured with exosomes as indicated in medium supplemented with EdU (10 µM, Thermo Fisher Scientific) for 24 h. Cells were fixed, permeabilized and processed for the Click-IT reaction following the manual. Cells were stained with DAPI (Beyotime) and captured using a confocal microscope (Nikon). The EdU positive rate (%) = the number of positive cells/total cells ×100%.

### Transwell invasion assay

Cell invasion was assessed by transwell assays with transwell chambers (8-µm pore membranes, Corning, NY, USA) precoated with Matrigel (Corning). Cells (1 × 10^6^ cells) were seeded on the upper chamber and cocultured with macrophages or exosomes as indicated. Cells which invaded into the lower chamber were fixed, stained with crystal violet (Sigma) and observed under the BX51 microscope (Olympus).

### RNA immunoprecipitation (RIP)

RIP assays were performed using the RIP kit (Millipore, Burlington, MA, USA). Magnetic beads were coated with the rabbit anti-Ago2 (ab186733, Abcam) or normal IgG (2729, Cell Signaling Technology, Danvers, MA, USA). A2780 and SKOV3 cells were lysed, and lysates were harvested. Magnetic beads and lysates were mixed well and incubated overnight. RNA was recovered and subjected to RT-qPCR.

### RNA pull-down

A2780 and SKOV3 cells were lysed and centrifugated for collecting cell lysates. Biotinylated *circTMCO3* probe or control oligo probe was mixed with cell lysates and incubated for 16 h at 4 °C. Streptavidin magnetic beads (Thermo Fisher Scientific) were added, mixed well, and incubated for additional 2 h with gentle rotation. Subsequently, RNA was recovered, and the abundance of miR-515-5p was examined by RT-qPCR.

### Dual-luciferase reporter assay

Wildtype and mutant binding sites for *miR-515-5p* in *circTMCO3* (circTMCO3-WT and circTMCO3-MUT) and the 3′ untranslated region (3′ UTR) of *ITGA8* (ITGA8-WT and ITGA8-MUT) were inserted into pmirGLO (Promega, Madison, WI, USA). A2780 and SKOV3 cells were cotransfected with luciferase reporters and *miR-515-5p* mimics or inhibitor. Cells were collected for determining the luciferase activity with the Dual-Glo Luciferase Assay System (Promega).

### An intraperitoneal xenograft mouse model of ovarian cancer

Twenty female BALB/c nude mice (five-week-old, Hunan SJA Laboratory Animal, Changsha, China) were randomly divided into four groups (*n* = 5 per group): Control, Exo, Exo-shNC, Exo-shcircTMCO3#1 and Exo-shcircTMCO3#2. SKOV3 cells (1 × 10^6^ cells in 200 µL of PBS) were injected intraperitoneally into nude mice. Subsequently, exosomes (30 µg in 200 µL of PBS) from M2, shNC or shcircTMCO3-transfected M2 macrophages were intraperitoneally injected into mice every three days. Control mice received 200 µL of PBS without exosomes. Mice were sacrificed through deep anesthesia with sodium pentobarbital after 21 days. The investigator was blinded to the group allocation during the experiment. Animal procedures were approved by the Animal Care and Use Committee of Hunan Cancer Hospital.

### Immunohistochemistry (IHC) staining

Tumors from mice and ovarian cancer and normal adjacent tissues were process for paraffin embedding and cut into 5-µm slices. Antigen retrieval was performed in pH 6.0 antigen retrieval solution (Invitrogen), and slices were blocked consecutively in H_2_O_2_ and BSA solution. Slides were incubated with anti-CD206 (1:50, ab64693, Abcam), anti-Ki67 (1:50, ab833, Abcam) or ITGA8 (1:100, ab243027, Abcam) overnight. Subsequently, slides were washed and incubated with a donkey anti-rabbit or mouse HRP-conjugated secondary antibody, and DAB substrate (Beyotime) was added. Slides were stained with hematoxylin and imaged with the BX51 microscope (Nikon).

### Statistics and reproducibility

Data from three independent experiments were presented as mean ± standard deviation. Kaplan–Meier curve was applied to analyze patient survival, and the survival time was statistically compared using the log rank test. The Pearson correlation was used to for correlation analysis of gene expression. A priori power analysis (G*Power software) was performed to assess sample size required to generate 80% power for detecting a significant (*P* < 0.05) effect of treatment. The normality of data was evaluated by the Shapiro–Wilk test. Considering the significance level of 5%, there were no significant deviations from the normality of all data (*P* > 0.05). The variance of groups was analyzed by the Student’s *t* test (two tailed) and one-way analysis of variance (ANOVA) with Turkey post hoc test. *P* < 0.05 was statistically significant. *P < 0.05, **P < 0.01 and ***P < 0.001.

### Reporting summary

Further information on research design is available in the Nature Portfolio Reporting Summary linked to this article.

### Supplementary information

Supplementary Information.

Description of Additional Supplementary Files

Supplementary data 1

Reporting Summary

## Data Availability

All data generated or analyzed during this study are included in this article. The datasets used and/or analyzed during the current study are available from the corresponding author on reasonable request. The source data behind the graphs in the paper can be found in Supplementary Data 1.
