# Supplementary material for: M2 macrophage-derived exosomal circTMCO3 acts through miR-515-5p and ITGA8 to enhance malignancy in ovarian cancer
Source: Commun Biol. 2024 May 16;7:583. doi: 10.1038/s42003-024-06095-8 (PMC11098810; doi:10.1038/s42003-024-06095-8)
Supplement: Supplementary file 1 — Supplementary Information. [file 42003_2024_6095_MOESM1_ESM.pdf]

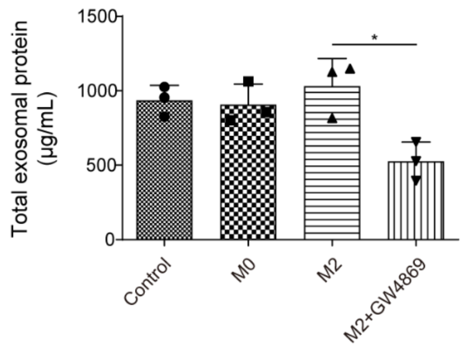

Supplementary Figure 1. Total exosomal protein was quantified through the BCA method in M0 and M2 macrophages derived from THP-1 cells treated with or without GW4869 (n=3). GW4869 was used to block exosome generation. * $P < 0.05$. Data were presented as mean ± standard deviation.

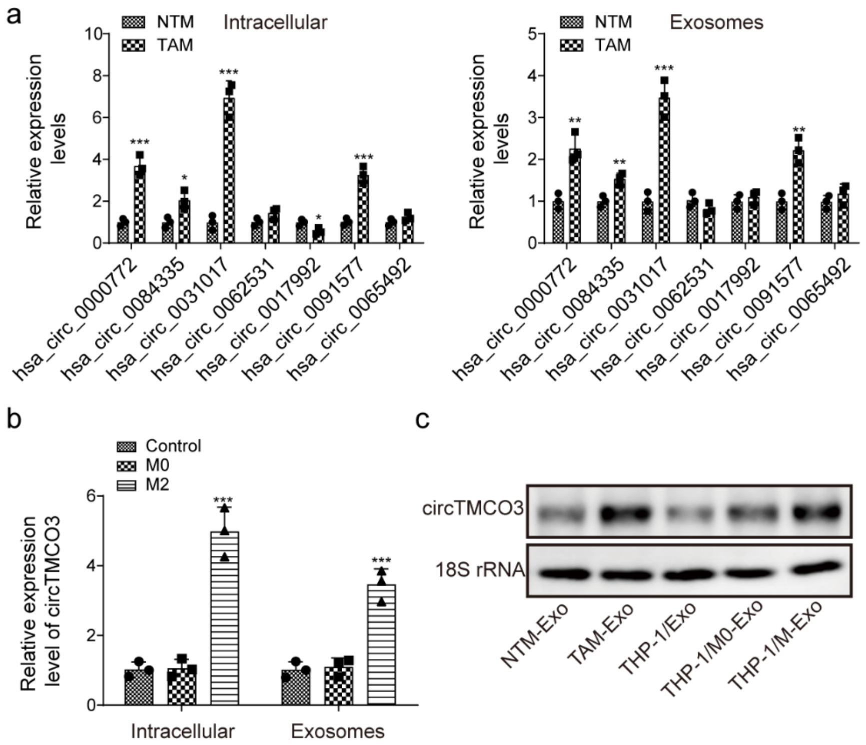

Supplementary Figure 2. CircTMCO3 expression was determined with RT-qPCR and Northern blot. (a) The expression of *hsa_circ_0000772, hsa_circ_0084335, hsa_circ_0031017, hsa_circ_0062531, hsa_circ_0017992, hsa_circ_0091577* and *hsa_circ_0065492* in TAMs, NTMs and exosomes derived from TAMs and NTMs was examined with RT-qPCR (n = 3). (b) The expression of

circTMCO3 in Control, M0 and M2 macrophages derived from THP-1 cells was determined with RT-qPCR (n = 3). (c) The expression of *circTMCO*3 in exosomes derived from NTMs, TAMs, THP-1 cells, M0 and M2 macrophages was examined via Northern blot. * P < 0.05, ** P < 0.01 and *** P < 0.001. Data were presented as mean ± standard deviation.

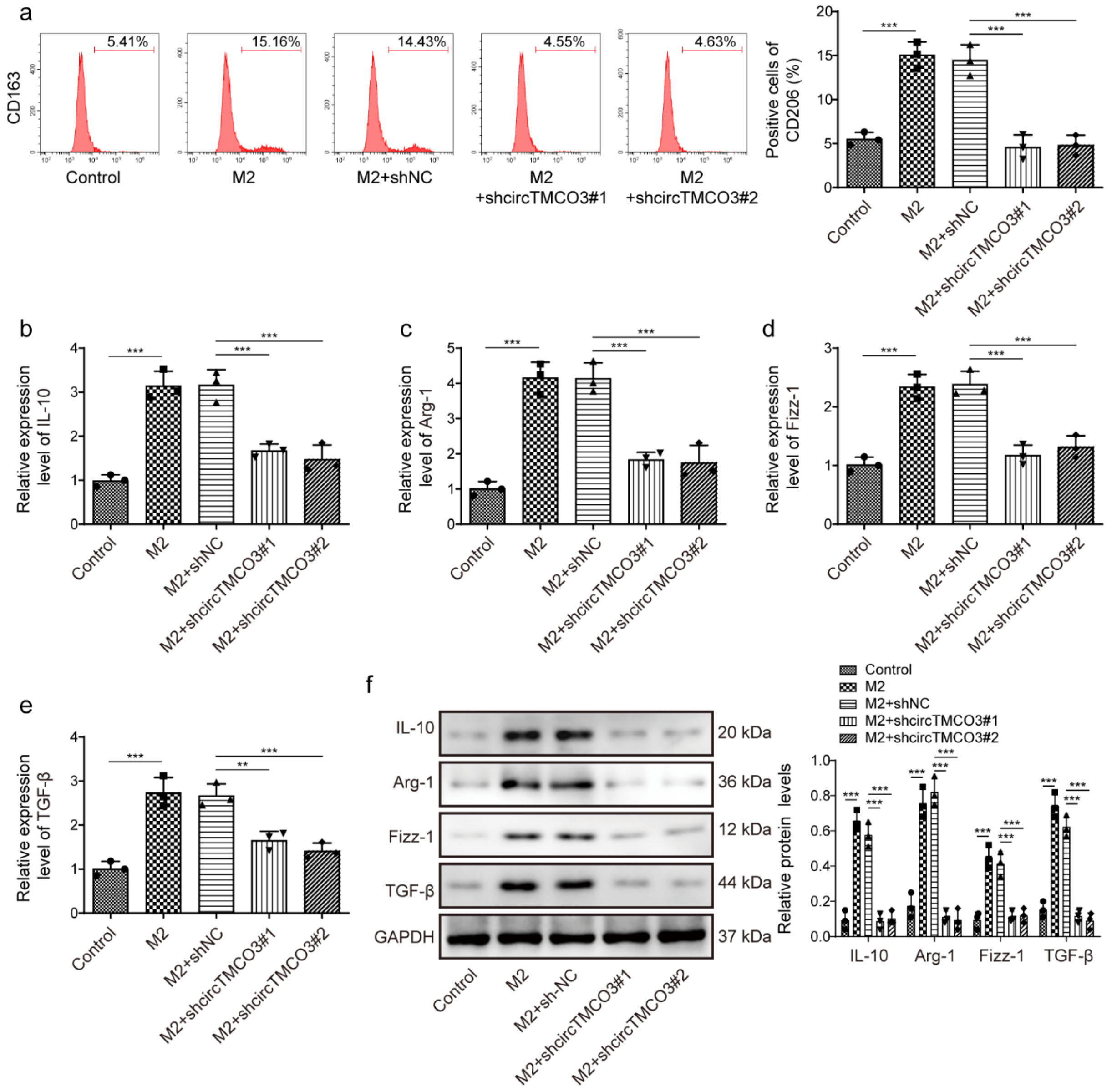

Supplementary Figure 3. Inhibition of circTMCO3 suppressed M2 macrophages. THP-1 cells were induced for M2 polarization and transfected with shNC or shcircTMCO3. (a) The ratio of CD163-

positive macrophages was analyzed by flow cytometry (n = 3). (b-f) RT-qPCR and Western blotting analysis of IL-10, Arg-1, Fizz-1 and TGF-β (n = 3). ** $P < 0.01$ and *** $P < 0.001$.

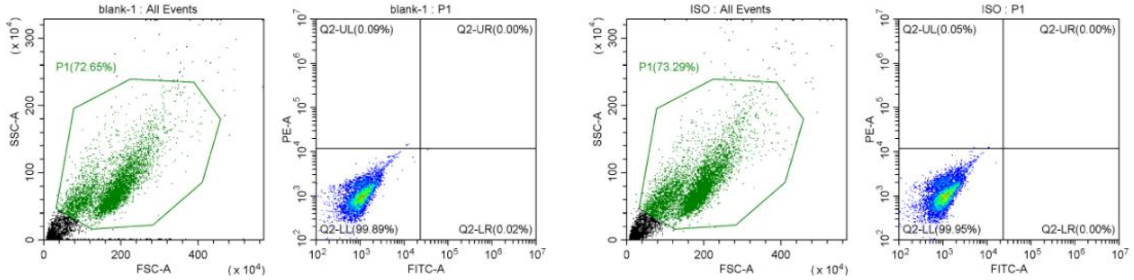

Supplementary Figure 4. The gating strategy for flow cytometry analysis in Figure 1B was provided.

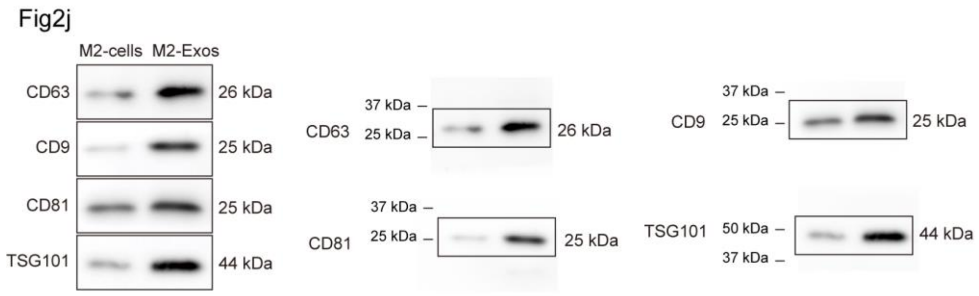

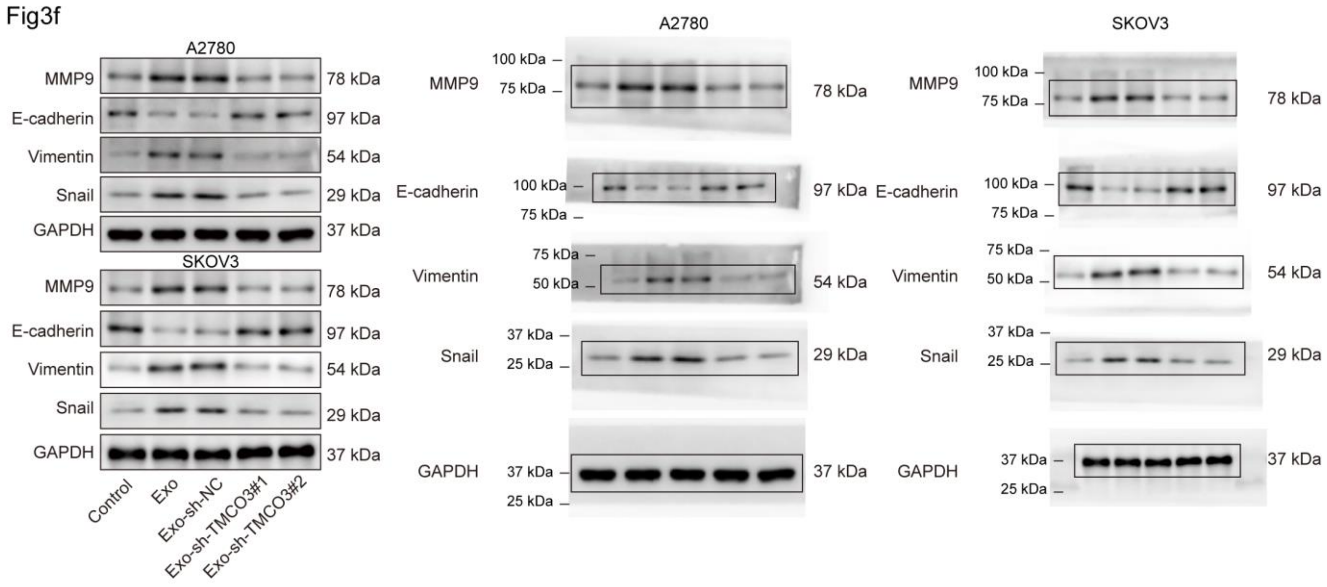

Fig6c

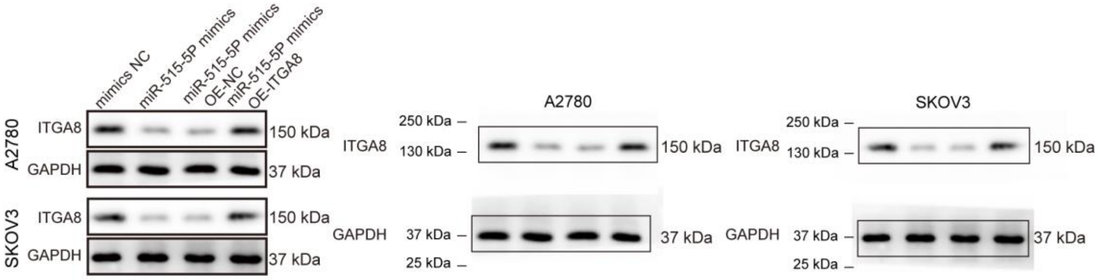

Fig6g

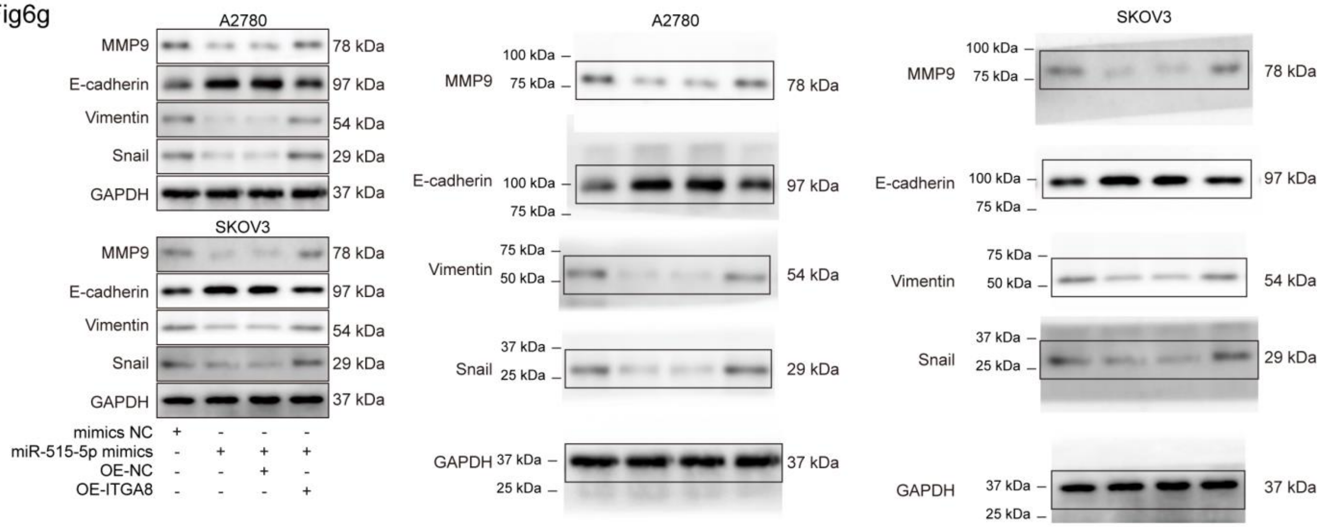

Fig7d

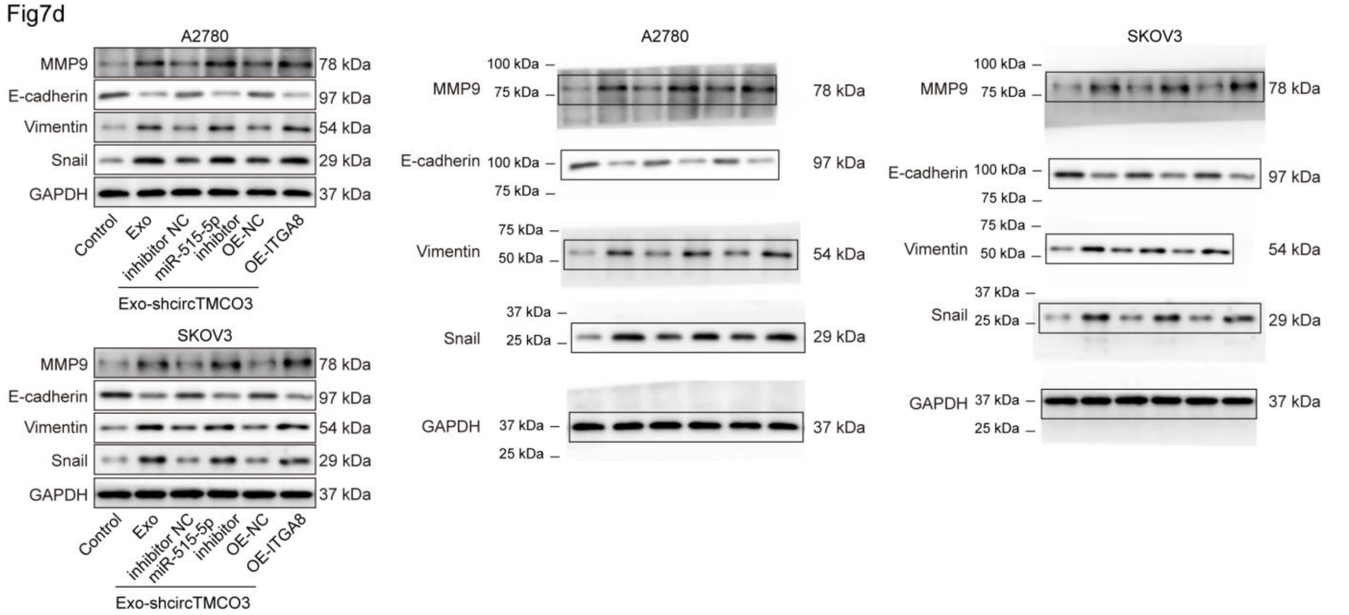

Fig8h

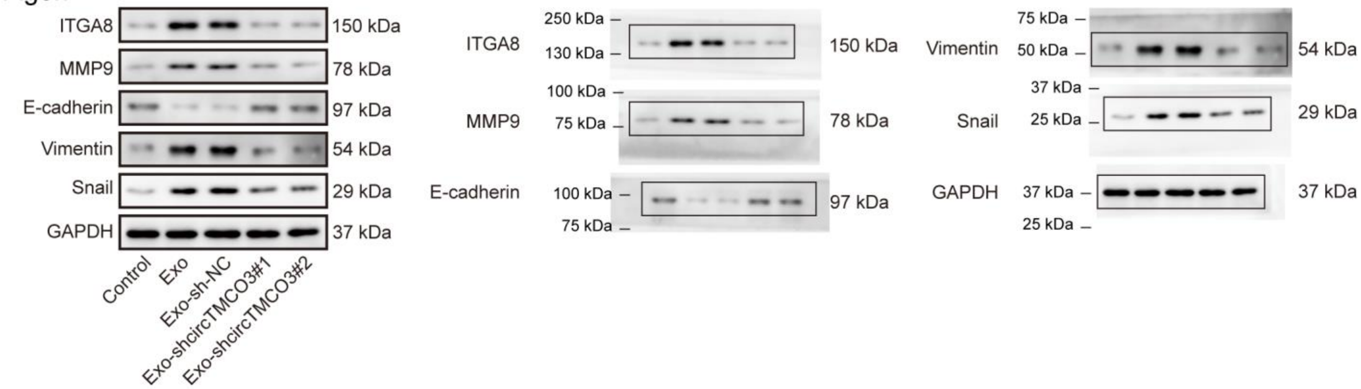

Fig3f

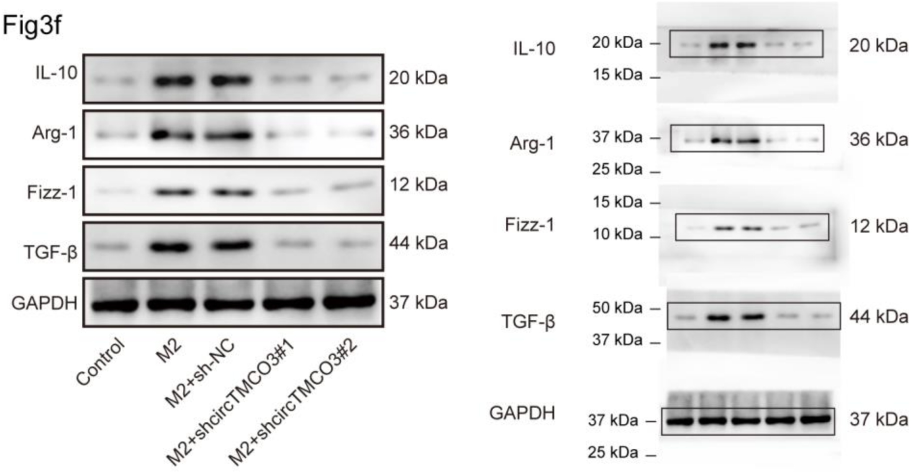

Supplementary Figure 5. The original and unedited western blot images.

Supplementary Table 1. RT-qPCR primers.

CircTMCO3	Forward: 5’-CATCTCTTGCGGATCAAACCC-3’
	Reverse: 5’-CACAGCAATCCACGGGTCTC-3’
TMCO3	Forward: 5’-GCTGATTGACTCCCAGAACAACC-3’
	Reverse: 5’-CCACAAGGCAAGGACAGCATTC-3’
TNF-α	Forward: 5’-ATGAGCACTGAAAGCATGATCC-3’
	Reverse: 5’-GAGGGCTGATTAGAGAGAGGTC-3’
iNOS	Forward: 5’-GTTCTCAAGGCACAGGTCTC-3’
	Reverse: 5’-GCAGGTCACTTATGTCACTTATC-3’
IL-10	Forward: 5’-AAGACCCAGACATCAAGGCG-3’
	Reverse: 5’-AATCGATGACAGCGCCGTAG-3’

Arg-1	Forward: 5’-GGCAAGGTGATGGAAGAAAC-3’
	Reverse: 5’-AGTCCGAAACAAGCCAAGGT-3’
miR-515-5p	Forward: 5’-GCGTTCTCCAAAAGAAAGCAC-3’
	Reverse: 5’-GTCGTATCCAGTGCAGGGTCC GAGGTATTCGCACTGGATACGACCAGAAA-3’
ITGA8	Forward: 5’-GCTGCTGGGGAGTTTACTGG-3’
	Reverse: 5’-GATGCCATCTGTTCTCCCGTG-3’
U6	Forward: 5’-AACGAGACGACGACAGAC-3’
	Reverse: 5’-GCAAATTCGTGAAGCGTTCCATA-3’
GAPDH	Forward: 5’-GGATTTGGTCGTATTGGG-3’
	Reverse: 5’-GGAAGATGGTGATGGGATT-3’
