# Supplementary material for: M2 macrophage-derived exosomal circTMCO3 acts through miR-515-5p and ITGA8 to enhance malignancy in ovarian cancer
Source: Commun Biol. 2024 May 16;7:583. doi: 10.1038/s42003-024-06095-8 (PMC11098810; doi:10.1038/s42003-024-06095-8)
Supplement: Supplementary file 4 — Reporting Summary [file 42003_2024_6095_MOESM4_ESM.pdf]

nature portfolio

Corresponding author(s): Ke-Qiang Zhang

Last updated by author(s): Feb 22, 2024

Reporting Summary

Nature Portfolio wishes to improve the reproducibility of the work that we publish. This form provides structure for consistency and transparency in reporting. For further information on Nature Portfolio policies, see our Editorial Policies and the Editorial Policy Checklist.

Statistics

For all statistical analyses, confirm that the following items are present in the figure legend, table legend, main text, or Methods section.

n/a	Confirmed	
☐	☒	The exact sample size (*n*) for each experimental group/condition, given as a discrete number and unit of measurement
☒	☐	A statement on whether measurements were taken from distinct samples or whether the same sample was measured repeatedly
☐	☒	The statistical test(s) used AND whether they are one- or two-sided<br>*Only common tests should be described solely by name; describe more complex techniques in the Methods section.*
☒	☐	A description of all covariates tested
☐	☒	A description of any assumptions or corrections, such as tests of normality and adjustment for multiple comparisons
☐	☒	A full description of the statistical parameters including central tendency (e.g. means) or other basic estimates (e.g. regression coefficient) AND variation (e.g. standard deviation) or associated estimates of uncertainty (e.g. confidence intervals)
☐	☒	For null hypothesis testing, the test statistic (e.g. *F*, *t*, *r*) with confidence intervals, effect sizes, degrees of freedom and *P* value noted<br>*Give P values as exact values whenever suitable.*
☒	☐	For Bayesian analysis, information on the choice of priors and Markov chain Monte Carlo settings
☒	☐	For hierarchical and complex designs, identification of the appropriate level for tests and full reporting of outcomes
☐	☒	Estimates of effect sizes (e.g. Cohen's *d*, Pearson's *r*), indicating how they were calculated

Our web collection on statistics for biologists contains articles on many of the points above.

Software and code

Policy information about availability of computer code

Data collection	No software was used.
Data analysis	No software was used.

For manuscripts utilizing custom algorithms or software that are central to the research but not yet described in published literature, software must be made available to editors and reviewers. We strongly encourage code deposition in a community repository (e.g. GitHub). See the Nature Portfolio guidelines for submitting code & software for further information.

Data

Policy information about availability of data

All manuscripts must include a data availability statement. This statement should provide the following information, where applicable:

- Accession codes, unique identifiers, or web links for publicly available datasets
- A description of any restrictions on data availability
- For clinical datasets or third party data, please ensure that the statement adheres to our policy

Provide your data availability statement here.

Research involving human participants, their data, or biological material

Policy information about studies with human participants or human data. See also policy information about sex, gender (identity/presentation), and sexual orientation and race, ethnicity and racism.

Reporting on sex and gender	N/A
Reporting on race, ethnicity, or other socially relevant groupings	N/A
Population characteristics	N/A
Recruitment	Sixty patients were diagnosed with ovarian cancer at Hunan Cancer Hospital, and tumor and adjacent normal tissues were collected and stored for RNA extraction and immunohistochemistry staining.
Ethics oversight	The Ethics Committee of Hunan Cancer Hospital approved this study.

Note that full information on the approval of the study protocol must also be provided in the manuscript.

Field-specific reporting

Please select the one below that is the best fit for your research. If you are not sure, read the appropriate sections before making your selection.

☒ Life sciences ☐ Behavioural & social sciences ☐ Ecological, evolutionary & environmental sciences

For a reference copy of the document with all sections, see nature.com/documents/nr-reporting-summary-flat.pdf

Life sciences study design

All studies must disclose on these points even when the disclosure is negative.

Sample size	A priori power analysis (G*Power software) was performed to estimate sample size required to generate 80% power for detecting a significant ($p < 0.05$) effect of treatment.
Data exclusions	N/A
Replication	All experiments were conducted with three replicateds, and each experiment was performed in triplicate.
Randomization	All mice were randomly divided into four groups.
Blinding	The investigator was binded to the group allocation during the experiment.

Reporting for specific materials, systems and methods

We require information from authors about some types of materials, experimental systems and methods used in many studies. Here, indicate whether each material, system or method listed is relevant to your study. If you are not sure if a list item applies to your research, read the appropriate section before selecting a response.

Materials & experimental systems

n/a	Involved in the study	
☐	☒	Antibodies
☐	☒	Eukaryotic cell lines
☒	☐	Palaeontology and archaeology
☐	☒	Animals and other organisms
☒	☐	Clinical data
☒	☐	Dual use research of concern
☒	☐	Plants

Methods

n/a	Involved in the study	
☒	☐	ChIP-seq
☐	☒	Flow cytometry
☒	☐	MRI-based neuroimaging

Antibodies

Antibodies used	The supplier name and catalog number of antibodies were provided in the manuscript.

Validation | N/A

Eukaryotic cell lines

Policy information about cell lines and Sex and Gender in Research

Cell line source(s)	A2780 and SKOV3 were purchased from iCell Bioscience Inc (Shanghai, China) and Cell Bank, Chinese Academy of Sciences (Shanghai, China), respectively. THP-1 was bought from the American Type Culture Collection (ATCC, Manassas, VA, USA).
Authentication	All cell lines were authenticated by STR DNA profiling analysis.
Mycoplasma contamination	All cell lines were detected to be free of mycoplasma contamination.
Commonly misidentified lines (See ICLAC register)	N/A

Animals and other research organisms

Policy information about studies involving animals; ARRIVE guidelines recommended for reporting animal research, and Sex and Gender in Research

Laboratory animals	BALB/c nude mice (five-week-old; Female) were used in this study.
Wild animals	N/A
Reporting on sex	N/A
Field-collected samples	N/A
Ethics oversight	Animal procedures were approved by the Animal Care and Use Committee of Hunan Cancer Hospital.

Note that full information on the approval of the study protocol must also be provided in the manuscript.

Plants

Seed stocks	N/A
Novel plant genotypes	N/A
Authentication	N/A

Flow Cytometry

Plots

Confirm that:

☒ The axis labels state the marker and fluorochrome used (e.g. CD4-FITC).

☒ The axis scales are clearly visible. Include numbers along axes only for bottom left plot of group (a 'group' is an analysis of identical markers).

☒ All plots are contour plots with outliers or pseudocolor plots.

☒ A numerical value for number of cells or percentage (with statistics) is provided.

Methodology

Sample preparation	Macrophages were isolated from tumor (TAM) and normal (NTM) tissues.
Instrument	Beckman cytoflex flow cytometry
Software	The Cytexpert Software

Cell population abundance	Abundance of CD163 and CD206 positive cell population
Gating strategy	Based on isotype group

☒ Tick this box to confirm that a figure exemplifying the gating strategy is provided in the Supplementary Information.
